# Psychological impact of body mass index changes in older adults: a 12-year longitudinal study

**DOI:** 10.1186/s12877-026-08050-0

**Published:** 2026-07-29

**Authors:** Yi-Chang Chou, Feng-Shiang Cheng, Shih-Han Weng, Chih-Hao Tseng, Hsiao-Yun Hu, Chieh-Hsing Liu

**Affiliations:** 1 Department of Health Promotion and Health Education, National Taiwan Normal University, No. 162, Sec. 1, Heping E. Rd., Da’an Dist., Taipei, 106209 Taiwan; 2 Department of Education and Research, Taipei City Hospital, Taipei, Taiwan; 3 University of Taipei, Taipei, Taiwan; 4 Institute of Public Health, National Yang Ming Chiao Tung University, Taipei, Taiwan; 5 Institute of Environmental Engineering and Management, National Taipei University of Technology, Taipei, Taiwan

**Keywords:** Body mass Iindex (BMI), Psychological distress, Older adults, Weight changes

## Abstract

**Background:**

Significant changes in body mass index (BMI) may be associated with psychological distress, particularly in older adults. However, few longitudinal studies have examined this relationship over time. This study aimed to investigate the association between BMI changes and psychological distress assessed repeatedly among community-dwelling older adults in Taiwan.

**Methods:**

This 12-year prospective cohort study included 70,570 adults aged ≥ 65 years who participated in the Taipei City Government’s annual physical examination program between 2005 and 2016. BMI changes were classified relative to baseline (Definition 1) and prior follow-up measurements (Definition 2). Psychological distress was assessed using the Brief Symptom Rating Scale (BSRS-5). Multivariable generalized estimating equation (GEE) models were used to evaluate associations between BMI changes and psychological distress. Sensitivity analyses included a Cox proportional-hazards model treating incident psychological distress (first BSRS-5 ≥ 6) as the outcome, with BMI change as a time-varying covariate, and a lagged model relating BMI change to distress at the subsequent examination.

**Results:**

Under both definitions, participants with major weight loss (> 10%) had the highest risk of psychological distress (Definition 1: adjusted odds ratio [aOR] = 1.90, 95% confidence interval [CI]: 1.77–2.04; Definition 2: aOR = 1.96, 95% CI: 1.80–2.14). Minor weight loss (5%–10%) also increased the risk (Definition 1: aOR = 1.60, 95% CI: 1.52–1.68; Definition 2: aOR = 1.52, 95% CI: 1.44–1.60). Minor and major weight gains were associated with elevated but comparatively lower risks. In sensitivity analyses, the association between weight loss and psychological distress remained significant and dose-dependent, although attenuated; associations with weight gain were not robust.

**Conclusion:**

Weight loss was consistently and dose-dependently associated with psychological distress in older adults and may serve as an early marker of declining health. Regular monitoring of weight change, rather than static weight status, may help prompt timely mental-health screening and nutritional or psychosocial assessment in this population.

**Supplementary Information:**

The online version contains supplementary material available at https://doi.org/10.1186/s12877-026-08050-0.

## Background

The global aging population is increasing, with one-sixth of the world expected to be aged ≥ 60 years by 2030. Over 14% of older adults suffer from psychological distress, with depression and anxiety being the most prevalent conditions, and being frequently overlooked or mistaken as a natural part of aging [1]. Older adults with mental health issues face reduced life satisfaction [2], an increased risk of cerebrovascular diseases [3], and mortality [4]. Given the significant health impacts of mental health problems, the need is urgent to identify risk factors for psychological distress early to safeguard the mental health of older adults.

Obesity and being overweight are also major public health challenges owing to their association with serious comorbidities, such as hypertension, diabetes, and heart disease [5], as well as psychological distress, including depression and anxiety symptoms [6]. While there is extensive research on the relationship between obesity and psychological distress in younger and middle-aged populations, studies focusing on older adults remain limited, and those that focus on this population present inconsistent evidence. A meta-analysis of observational studies involving 171,701 adults demonstrated that individuals with obesity were 32% more likely to have depression compared to those with normal weight [6]. However, a meta-analysis of studies on community-dwelling older adults revealed an inverse relationship, with overweight and obese older adults being significantly less likely to have depression compared to their normal-weight counterparts [7]. These findings support the “jolly fat” hypothesis, suggesting that higher BMI in older adults may confer mental health benefits, possibly owing to greater functional reserve or social factors. These conflicting results highlight the importance of investigating the unique dynamics of BMI and psychological distress in older adults, as the mechanisms and implications may differ significantly from those observed in younger populations.

Besides static weight status, weight changes among older individuals often reflect declines in muscle mass and bone density [8], and have been associated with increased mortality [9, 10], reduced health-related quality of life [11], and cognitive impairment [12, 13]. However, research on the association between weight change and psychological distress remains limited and findings inconsistent. For instance, a large meta-analysis of 15 studies reported no significant association between weight change and depression [14]. Recently, a study focusing on middle-aged and older adults found that weight loss exceeding 3% was associated with a higher risk of depressive symptoms than stable weight [15]. Further research is thus warranted to determine whether weight change effectively predicts psychological distress among older adults. In particular, prior studies have largely relied on static or baseline body weight, examined depression rather than broader psychological distress, been conducted in clinical or middle-aged populations, and used a single metric of weight change, leaving us with a lack of clarity as to the prospective associations in community-dwelling older adults.

To address these gaps, we conducted a 12-year longitudinal prospective cohort study using data from community-dwelling older adults in Taiwan to examine the association between BMI changes, including weight loss and gain, and psychological distress assessed repeatedly during follow-up. Moreover, this study examined both short-term BMI changes (compared to the most recent BMI) and long-term cumulative BMI changes (compared to baseline BMI across multiple follow-ups).

## Methods

### Study population

**Fig. 1:**
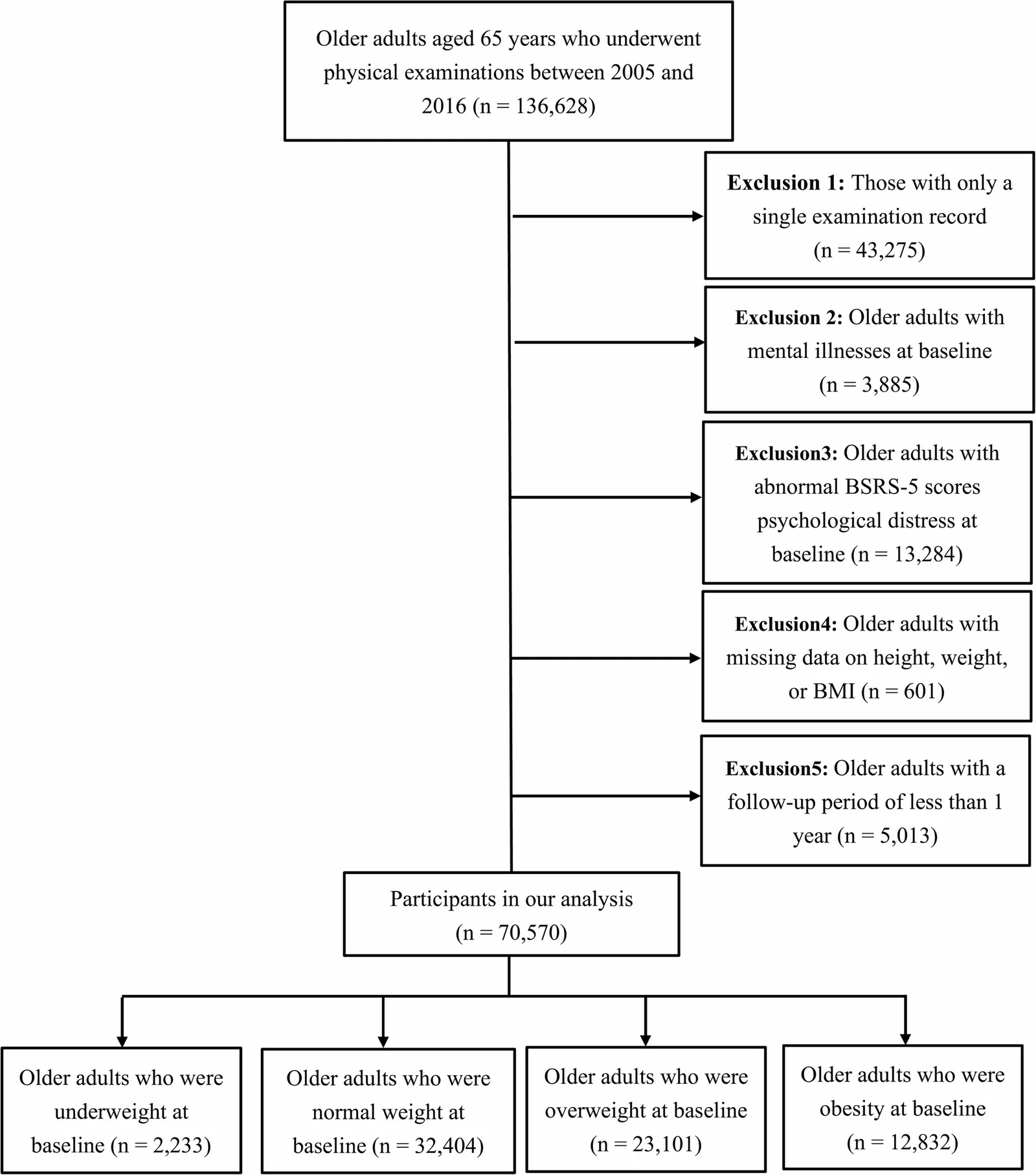
Flow chart of the patient selection process

The data for this 12-year prospective longitudinal cohort study were obtained from the Taipei City Elderly Health Examination Database. The study population included older adults who participated in a complimentary annual physical examination program organized by the Taipei City Government from May 1, 2005, to December 31, 2016. A total of 136,628 participants aged 65 years and above were enrolled. These individuals voluntarily underwent routine health check-ups and were encouraged to participate annually. The assessments comprised standardized medical evaluations and self-reported questionnaires addressing various health-related aspects, including psychological distress screening. A total of 70,570 participants were included in the final analysis after excluding individuals with only a single examination record (*n* = 43,275), those diagnosed with mental illnesses (*n* = 3,885), those with abnormal Brief Symptoms Rating Scale (BSRS-5) scores indicative of psychological distress (*n* = 13,284) at baseline, and older adults with missing data on height, weight, or BMI (*n* = 601). Excluding participants with distress at baseline yielded a distress-free cohort at entry, reducing reverse causality and ensuring that BMI changes during follow-up preceded any subsequent distress. Furthermore, participants with a follow-up period of less than one year (*n* = 5,013) were excluded (Fig. 1 shows a flowchart of the sample selection process). Mental illnesses were determined based on participants’ self-reported personal history of mental illness or the use of psychotropic or sleeping medications as indicated in the baseline questionnaire. All participant identification data were encrypted to ensure confidentiality before analysis. The study was conducted per the principles outlined in the Declaration of Helsinki and was approved by the Institutional Review Board of Taipei City Hospital (IRB No. TCHIRB-11303014-W).

### Psychological distress

Psychological distress was evaluated during each physical examination using the BSRS-5 developed by Lee et al. [16]. Participants were asked to report their mental health state over the previous week. The BSRS-5 includes five self-reported items, as follows: (1) difficulty falling asleep (insomnia), (2) feeling tense or anxious (anxiety), (3) feeling easily annoyed or irritated (hostility), (4) feeling low in mood (depression), and (5) feeling inferior to others (inferiority). Each item is scored on a scale of 0 to 4, with 0 indicating “not present” and 4 indicating “extremely severe,” resulting in a total score ranging from 0 to 20. A total score of ≥ 6 was used as the cut-off point for identifying psychological distress, suggesting the potential presence of a psychiatric condition and the need for further evaluation, while a score ≤ 5 were classified as normal. The BSRS-5 is widely used in Taiwan as a rapid screening tool for psychological distress, with established validity and reliability in the general community population [16, 17]. Additionally, previous studies have applied the BSRS-5 in health examinations for older adults [18, 19].

### BMI classification and definition of BMI changes

Body mass index (BMI, kg/m²) was calculated using a standardized process: weight in kilograms divided by height in meters squared. Height and weight were measured at each examination using a standardized protocol, and BMI was recalculated at every visit. BMI classification followed the guidelines of Taiwan’s Ministry of Health and Welfare, which define underweight as BMI < 18.5 kg/m², normal weight as 18.5 ≤ BMI < 24 kg/m², overweight as 24 ≤ BMI < 27 kg/m², and obesity as BMI ≥ 27 kg/m² [20]. These criteria for BMI have been widely used in previous studies conducted in Taiwan [21, 22]. BMI changes during the follow-up period were defined in two ways: (1) Definition 1: BMI changes were calculated relative to the baseline measurement at the start of the study; (2) Definition 2: BMI changes were calculated relative to the previous measurement in the study’s follow-up intervals. The changes were determined as the difference between the BMI at each follow-up examination (from the second examination onward) and the reference BMI (baseline or previous measurement) divided by the reference BMI. These changes were categorized into five groups: major weight loss (BMI change < − 10%), minor weight loss (− 10% ≤ BMI change < − 5%), stable weight status (− 5% ≤ BMI change < 5%), minor weight gain (5% ≤ BMI change < 10%), and major weight gain (BMI change ≥ 10%) [12, 13]. These categories were used to examine the association between BMI fluctuations and psychological distress over time.

### Covariates

Covariates for this study were selected based on prior research [18, 21]. Demographic characteristics included age (years), sex (male or female), marital status (married or others), and co-residence status (living alone or with others). Socioeconomic status variables comprised educational attainment (≤ 6 years, 7–9 years, or ≥ 10 years) and low-income household status (yes or no). Health-related behaviors encompassed smoking status (current smoker or nonsmoker), alcohol consumption (occasional or frequent drinker, or never), and exercise (≥ 20 min per week; yes or no). Health conditions, including a history of chronic disease medication use or self-reported medical history (hypertension, diabetes, hyperlipidemia, heart disease, and stroke; yes or no), were assessed via self-administered questionnaires. Demographic characteristics (excluding sex), socioeconomic status, health-related behaviors, and health conditions were measured during each examination and treated as time-varying variables. In Taiwan, older adults in this cohort were educated under an educational system where compulsory education was limited to primary school (6 years). Further education required entrance examinations, making 7–9 years (junior high school) and ≥ 10 years (senior high school or above) significant milestones. Low-income households in Taiwan are defined as those with an average monthly disposable income per person at or below 60% of the minimum standard of living expense (≤ 19,013 New Taiwan Dollars [NTD]). Eligibility for low-income household status is reviewed annually by the Taipei City Government [23].

### Statistical analyses

Participants’ baseline characteristics across different BMI categories were compared using Analysis of Variance (ANOVA) for continuous variables and the chi-square test for categorical variables, as appropriate. Longitudinal relationships between BMI changes during follow-up and the presence of psychological distress were analyzed using generalized estimating equations (GEEs). The analysis was performed using three models: (1) Model 1, univariable analysis; (2) Model 2, adjusted for age and sex; (3) Model 3, further adjusted for health examination visits, years of education, low-income household status, marital status, living arrangement, smoking status, drinking status, exercise habits, baseline BMI, and comorbidities including hypertension, heart disease, diabetes, and hyperlipidemia. The multivariable adjustment in Models 2 and 3 allowed for a more comprehensive control of potential confounders, enabling the evaluation of the independent association between BMI categories, BMI changes, and psychological distress, respectively.

GEEs accounted for cumulative longitudinal data and managed repeated measurements across follow-up periods, with one observation per participant per examination. This approach enables the identification of overall trends throughout the study period and is widely utilized in longitudinal studies involving repeated measures [18, 21]. In the primary analysis, psychological distress and BMI changes were treated as categorical variables within the GEEs model, with the presence of psychological distress (BSRS-5 ≥ 6) at each examination as the outcome; the model estimated the population-averaged association between BMI change and psychological distress across repeated examinations, accounting for within-person correlation through an autoregressive (AR-1) working correlation structure. Subgroup analysis was performed to investigate whether the association between BMI changes and psychological distress varied by age (65–79 years, ≥ 80 years), sex (male, female), and educational attainment (≤ 6 years, ≥ 7 years).

A logit link function was applied in the GEEs analysis. Odds ratios (OR) with 95% confidence intervals (CI) were calculated to evaluate the strength and direction of associations. Data management and statistical analyses were conducted using SAS software (version 9.4; SAS Institute, Cary, NC, USA) and R version 4.4.1 (ggplot2 and forestplot.

package). Statistical significance was defined as a two-tailed p-value of < 0.05.

We conducted two sensitivity analyses to address temporal relationships and incident-event framing. First, to model the first occurrence of psychological distress, we fitted a Cox proportional-hazards model with BMI change as a time-varying covariate, using years since the baseline examination (derived from examination dates) as the time scale and the first BSRS-5 ≥ 6 during follow-up as the event, with censoring at the last examination. Second, to ensure that the exposure preceded the outcome, we fitted a lagged model relating BMI change at a given examination to psychological distress at the subsequent examination, restricted to participants without distress at the index examination. Both analyses were adjusted for the same covariates as the primary model.

## Results

### Participant characteristics

Among the 70,570 participants, 51.3% were male, with a mean (standard deviation, SD) age of 71.9 (6.3) years at baseline. Educational attainment showed that 31.3% had ≤ 6 years of education, while 54.9% had ≥ 10 years. At baseline, BMI category distribution was as follows: 3.1% were classified as underweight, 46.0% had normal weight, 32.7% were overweight, and 18.2% were obese. As shown in Table 1, participants classified as underweight were typically older (mean ± SD: 73.8 ± 7.3 years), more likely to be female (52.8%), and had a higher prevalence of low-income household status (6.4%) compared to other BMI groups.

**Table 1:** Baseline characteristics and demographics among participants with different BMI categories at baseline (*N* = 70,570)

Characteristics, *n* (%)	Total	Underweight (*n* = 2,232)	Normal weight (*n* = 32,447)	Overweight (*n* = 23,058)	Obesity (*n* = 12,833)	*P*-value
Age, yrs (mean ± SD)	71.9 ± 6.3	73.8 ± 7.3	71.9 ± 6.4	71.8 ± 6.1	71.7 ± 6.0	< 0.001
Sex						
Male	36,190 (51.3)	1,053 (47.2)	16,211 (50.0)	12,795 (55.5)	6,131 (47.8)	< 0.001
Female	34,380 (48.7)	1,179 (52.8)	16,236 (50.0)	10,263 (44.5)	6,702 (52.3)	
Educational attainment (years), missing data = 1,327						
≤ 6	21,696 (31.3)	651 (29.6)	8,715 (27.4)	7,219 (31.9)	5,111 (40.6)	< 0.001
7–9	9,540 (13.8)	305 (13.9)	4,262 (13.4)	3,138 (13.9)	1,835 (14.6)	
≥ 10	38,007 (54.9)	1,241 (56.5)	18,871 (59.3)	12,264 (54.2)	5,631 (44.8)	
Low–income household	2,885 (4.1)	142 (6.4)	1,247 (3.9)	939 (4.1)	557 (4.3)	< 0.001
Married, missing data = 233	53,194 (75.6)	1,556 (69.8)	24,343 (75.2)	17,715 (77.1)	9,580 (75.0)	< 0.001
Living alone	4,054 (5.7)	167 (7.5)	1,974 (6.1)	1,220 (5.3)	693 (5.4)	< 0.001
Current smoker, missing data = 91	5,525 (7.8)	241 (10.8)	2,457 (7.6)	1,848 (8.0)	979 (7.6)	< 0.001
Occasional or frequent drinking, missing data = 103	14,145 (20.1)	299 (13.4)	6,114 (18.9)	5,133 (22.3)	2,599 (20.3)	< 0.001
Exercise (≥ 20 min per week), missing data = 269	64,711 (92.1)	1,976 (88.6)	29,952 (92.8)	21,294 (92.6)	11,489 (89.9)	< 0.001
Medication history						
Hypertension	26,437 (37.5)	420 (18.8)	9,783 (30.2)	9,628 (41.8)	6,606 (51.5)	< 0.001
Diabetes	7,295 (10.3)	98 (4.4)	2,841 (8.8)	2,521 (10.9)	1,835 (14.3)	< 0.001
Hyperlipidemia	6,629 (9.4)	148 (6.6)	2,899 (8.9)	2,276 (9.9)	1,306 (10.2)	< 0.001
Heart disease	11,972 (17.0)	328 (14.7)	4,874 (15.0)	4,090 (17.7)	2,680 (20.9)	< 0.001
Stroke	2,197 (3.1)	67 (3.0)	1,061 (3.3)	697 (3.0)	372 (2.9)	0.150

*BMI* Body mass index, *married* Married living together, cohabiting, *SD* Standard deviation

The average (SD) length of the long-term follow-up period was 5.7 (3.1) years (range, 1.0–11.6 years). The average interval between consecutive measurements for short-term BMI changes was 1.32 (0.87) years. A total of 4,229 (6.0%) out of 70,570 participants were identified as having psychological distress during their final examination. At the last follow-up, compared to baseline BMI, 4.9% of participants experienced major weight gain, 11.6% had minor weight gain, 61.7% maintained stable weight status, 14.0% experienced minor weight loss, and 7.8% had major weight loss (Table 2).

**Table 2:** BMI category, BMI change, and psychological distress at the last examination (*N* = 70,570)

Variable, *n* (%)	Total	BSRS-5 ≥ 6 (*n* = 4,229)	BSRS-5 < 6 (*n* = 66,341)	*P*-value
Follow-up duration (years, mean ± SD)	5.7 ± 3.1	5.6 ± 3.1	5.7 ± 3.1	0.224
BMI category				
Underweight	3,234 (4.6)	277 (6.6)	2,957 (4.5)	< 0.001
Normal weight	33,071 (46.9)	1,984 (46.9)	31,087 (46.9)	
Overweight	21,480 (30.4)	1,203 (28.5)	20,277 (30.6)	
Obesity	12,785 (18.1)	765 (18.1)	12,020 (18.1)	
Definition 1^a^				
BMI change				
Major weight gain	3,459 (4.9)	230 (5.4)	3,229 (4.9)	< 0.001
Minor weight gain	8,178 (11.6)	493 (11.7)	7,685 (11.6)	
Stable weight status	43,555 (61.7)	2,269 (53.7)	41,286 (62.2)	
Minor weight loss	9,889 (14.0)	723 (17.1)	9,166 (13.8)	
Major weight loss	5,489 (7.8)	514 (12.2)	4,975 (7.5)	
Definition 2^b^				
BMI change				
Major weight gain	1,901 (2.7)	166 (3.9)	1,735 (2.6)	< 0.001
Minor weight gain	5,907 (8.4)	397 (9.4)	5,510 (8.3)	
Stable weight status	53,117 (75.3)	2,872 (67.9)	50,245 (75.7)	
Minor weight loss	7,095 (10.1)	527 (12.5)	6,568 (9.9)	
Major weight loss	2,550 (3.6)	267 (6.3)	2,283 (3.4)	

*BMI* Body mass index, *BSRS-5* Brief Symptoms Rating Scale, *SD* Standard deviation. BMI change was classified as major weight loss (BMI change < − 10%), minor weight loss (− 10% ≤ BMI change < − 5%), stable weight status (− 5% ≤ BMI change < 5%), minor weight gain (5% ≤ BMI change < 10%), and major weight gain (BMI change ≥ 10%)

^a^Definition 1: BMI changes were defined relative to the baseline measurement at the start of the study

^b^Definition 2: BMI changes were defined relative to the previous measurement in the study’s follow-up intervals

### Association between BMI categories, BMI changes, and psychological distress

Table 3 presents the associations between BMI categories, BMI changes, and psychological distress based on multivariable-adjusted GEE analysis. Participants classified as underweight had a significantly higher risk of psychological distress (adjusted OR [aOR] = 1.35, 95% CI: 1.22–1.49), while those classified as overweight exhibited a decreased risk (aOR = 0.81, 95% CI: 0.77–0.85). Participants with obesity also showed a significantly lower risk (aOR = 0.78, 95% CI: 0.71–0.84) than those with normal weight.

**Table 3:** Association of BMI category, BMI change, and psychological distress by multivariable–adjusted generalized estimating equation analysis

Variable	Model 1^a^		Model 2^b^		Model 3^c^	
	OR	95% CI	OR	95% CI	OR	95% CI
BMI category						
Underweight	1.34^***^	1.23–1.47	1.23^***^	1.12–1.34	1.35^***^	1.22–1.49
Normal weight	Reference		Reference		Reference	
Overweight	0.87^***^	0.83–0.91	0.89^***^	0.86–0.93	0.81^***^	0.77–0.85
Obesity	0.97	0.92–1.03	0.98	0.92–1.03	0.78^***^	0.71–0.84
Definition 1^d^						
BMI change						
Major weight gain	1.63^***^	1.49–1.77	1.45^***^	1.33–1.59	1.29^***^	1.18–1.41
Minor weight gain	1.45^***^	1.37–1.53	1.37^***^	1.29–1.44	1.27^***^	1.20–1.35
Stable weight status	Reference		Reference		Reference	
Minor weight loss	1.79^***^	1.71–1.88	1.68^***^	1.61–1.77	1.60^***^	1.52–1.68
Major weight loss	2.36^***^	2.21–2.53	2.08^***^	1.95–2.23	1.90^***^	1.77–2.04
Definition 2^e^						
BMI change						
Major weight gain	1.70^***^	1.54–1.88	1.54^***^	1.40–1.70	1.51^***^	1.36–1.66
Minor weight gain	1.51^***^	1.42–1.59	1.43^***^	1.35–1.51	1.38^***^	1.30–1.46
Stable weight status	Reference		Reference		Reference	
Minor weight loss	1.66^***^	1.58–1.75	1.57^***^	1.49–1.65	1.52^***^	1.44–1.60
Major weight loss	2.27^***^	2.09–2.47	2.03^***^	1.87–2.21	1.96^***^	1.80–2.14

*BMI* Body mass index, *CI* Confidence intervals, *OR* Odds ratios. BMI change was classified as major weight loss (BMI change < − 10%), minor weight loss (− 10% ≤ BMI change < − 5%), stable weight status (− 5% ≤ BMI change < 5%), minor weight gain (5% ≤ BMI change < 10%), and major weight gain (BMI change ≥ 10%)

^a^Unadjusted

^b^Adjusted for age and sex

^c^Adjusted for age, sex, health examination visits, years of education, low-income household status, marital status, living alone, smoking, drinking, exercise habits, baseline body mass index (BMI), and chronic diseases (hypertension, heart disease, diabetes, and hyperlipidemia)

^d^Definition 1: BMI changes were defined relative to the baseline measurement at the start of the study

^e^Definition 2: BMI changes were defined relative to the previous measurement in the study’s follow-up intervals

^*^p < .05, ^**^ *p* < .01, ^***^ *p* < .001

For BMI changes under Definition 1, participants with major weight loss had the highest risk of psychological distress (aOR = 1.90, 95% CI: 1.77–2.04), followed by those with minor weight loss (aOR = 1.60, 95% CI: 1.52–1.68). Participants with minor (aOR = 1.27, 95% CI: 1.20–1.35) and major weight gain (aOR = 1.29, 95% CI: 1.18–1.41) also demonstrated significantly increased risks compared to those with stable weight status.

For BMI changes under Definition 2, major weight loss again showed the highest risk of psychological distress (aOR = 1.96, 95% CI: 1.80–2.14), followed by minor weight loss (aOR = 1.52, 95% CI: 1.44–1.60). Participants with minor (aOR = 1.38, 95% CI: 1.30–1.46) and major weight gain (aOR = 1.51, 95% CI: 1.36–1.66) also exhibited significantly increased risks compared to those with stable weight status.

### Subgroup analysis of BMI change and psychological distress

**Fig. 2:**
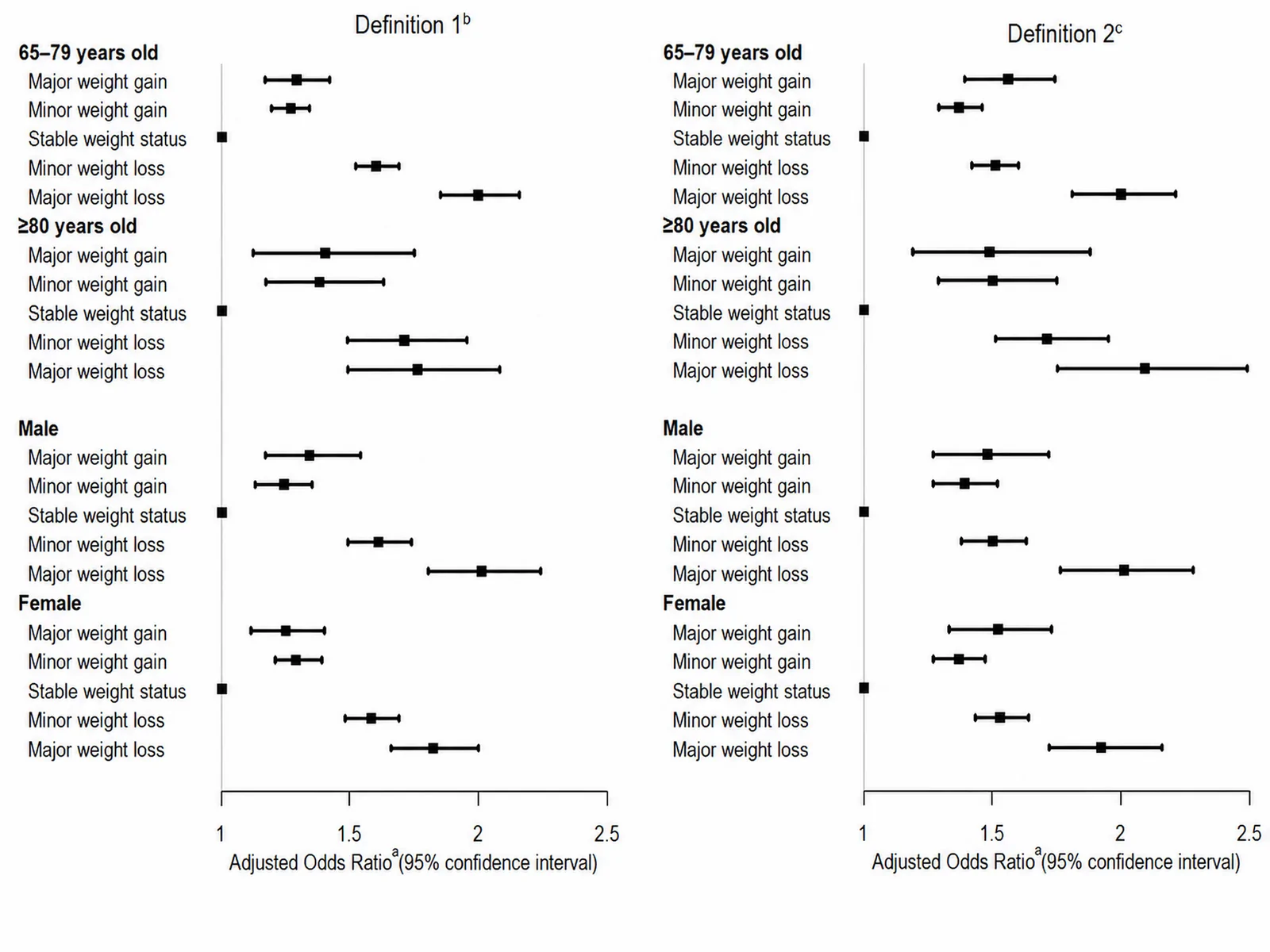
Subgroup analysis of the association between BMI changes and psychological distress among older adults, stratified by age and sex, using multivariable-adjusted generalized estimating equation analysis under two definitions of BMI changes. ^a^Adjusted for age, sex, health examination visits, years of education, low-income household status, marital status, living alone, smoking, drinking, exercise habits, baseline body mass index (BMI), and chronic disease (hypertension, heart disease, diabetes, and hyperlipidemia). ^b^Definition 1: BMI changes were defined relative to the baseline measurement at the start of the study. ^c^Definition 2: BMI changes were defined relative to the previous measurement in the study’s follow-up intervals

Figure 2 shows the stratified analysis of BMI changes (%) and their associations with psychological distress across subgroups based on age and sex. Under Definition 1, major weight loss consistently showed the highest risk of psychological distress in both age groups (65–79 years: aOR = 2.00, 95% CI: 1.85–2.16; ≥80 years: aOR = 1.76, 95% CI: 1.49–2.08), followed by minor weight loss. Weight gain, both major and minor, also posed an increased risk, though to a lesser extent.

Using Definition 2, major weight loss remained strongly associated with psychological distress, with a higher risk observed in the ≥ 80 years group (aOR = 2.09, 95% CI: 1.75–2.49) compared to the 65–79 years group (aOR = 2.00, 95% CI: 1.81–2.21).

For sex, major weight loss posed a higher risk for psychological distress in both men and women under both definitions. In Definition 1, men had an aOR of 2.01 (95% CI: 1.80–2.24), while women had an aOR of 1.82 (95% CI: 1.66–2.00). Under Definition 2, the risk associated with major weight loss was slightly higher in men (aOR = 2.01, 95% CI: 1.76–2.28) compared to women (aOR = 1.92, 95% CI: 1.72–2.16).

In the Cox proportional-hazards analysis, which modeled the first occurrence of psychological distress (10,840 incident events), weight loss remained associated with psychological distress in a dose-dependent manner under both definitions (Definition 1: minor weight loss adjusted hazard ratio [aHR] = 1.16, 95% CI: 1.10–1.23; major weight loss aHR = 1.25, 95% CI: 1.15–1.35; Definition 2: minor weight loss aHR = 1.07, 95% CI: 1.00–1.14; major weight loss aHR = 1.19, 95% CI: 1.08–1.31), although effect sizes were attenuated relative to the primary GEE model. In contrast, weight gain was not associated with an increased hazard of psychological distress and, for cumulative change from baseline, was inversely associated (Definition 1: minor weight gain aHR = 0.91, 95% CI: 0.85–0.96; major weight gain aHR = 0.85, 95% CI: 0.77–0.94). The lagged model, where BMI change preceded the distress assessment, showed the same pattern: weight loss predicted distress at the subsequent examination (Definition 1: minor weight loss aOR = 1.18, 95% CI: 1.11–1.26; major weight loss aOR = 1.32, 95% CI: 1.19–1.45), whereas associations with weight gain were null or smaller (Supplementary Tables S1 and S2).

## Discussion

This study utilized 12 years of longitudinal data to explore both short- and long-term BMI changes with psychological distress among community-dwelling older adults. The findings indicated that underweight was associated with higher psychological distress, whereas overweight and obese were associated with lower odds of psychological distress. Moreover, BMI changes, particularly weight loss, were consistently associated with higher odds of psychological distress, underscoring the importance of monitoring weight trajectories in older adults.

Previous studies have largely explored the relationship between baseline body weight and depression risk, but failed to pay due attention to the effects of weight changes on depression. Several investigations have focused on patients with diabetes-related conditions [24–26]. A Korean study reported that weight variability was associated with an increased risk of depression in individuals with type 2 diabetes mellitus, suggesting that weight fluctuations and fat accumulation may trigger inflammatory responses that contribute to depression [24]. Additionally, a large-scale study on patients with diabetes found a U-shaped relationship between weight changes and depression risk, with elevated risks observed in both the weight loss and weight gain groups compared to those with stable weight [26]. Similarly, a longitudinal study conducted in China among 3,118 middle-aged individuals demonstrated that weight loss exceeding 3% over 2–4 years was associated with a 1.3-fold higher risk of depression compared to stable weight [15]. However, these studies have important limitations, such as the inclusion of specific populations prone to fluid retention and edema, which may cause weight fluctuations [25], small sample sizes [15], and ambiguity in the direction of weight changes, whether gain or loss, when describing weight variability [24]. Moreover, none of these studies specifically targeted older adults aged 65 years or above.

Standing apart from these past studies, the current research demonstrated that both long-term and short-term BMI changes were associated with an increased risk of psychological distress, with weight loss posing a consistently higher risk than weight gain among older adults. These findings align with studies showing that weight changes, particularly weight loss, increase the risk of frailty and all-cause mortality [9, 10, 27], underscoring the potential role of psychological distress as an early indicator of underlying health issues.

The association between weight changes and psychological distress may involve immunometabolic dysregulation, including chronic low-grade inflammation, oxidative stress, and neuroendocrine disturbances such as insulin resistance [28]. Chronic inflammation, characterized by elevated markers such as C-reactive protein, often precedes weight changes and contributes to depressive symptoms [29, 30]. Additionally, dysregulation in the hypothalamic-pituitary-adrenal axis may exacerbate weight gain and loss, leading to changes in body composition, such as increased visceral fat and reduced lean mass, both of which are linked to depression [31–33]. These mechanisms highlight the complex pathways through which weight fluctuations may affect mental health.

This study utilized the BSRS-5 as a screening tool for psychological distress, which has demonstrated good sensitivity in identifying psychiatric morbidity [17]. While most existing studies on BMI and BMI changes in older adults have primarily focused on depression as the outcome, psychological distress encompasses a broader range of mental health disorders, including anxiety, hostility, and somatization [34]. Our study found that underweight older adults had a higher risk of psychological distress compared to those with normal weight, which aligns with most existing literature [15, 19, 35, 36]. One possible explanation involves low BMI in older adults potentially reflecting underlying health conditions, such as increased mortality risk [37], declines in muscle mass and bone density, or severe malnutrition [38]. Alternatively, depression or stress may suppress appetite, leading to insufficient nutrient intake and weight loss [39]. What this shows is how multifaceted is the relationship between low BMI and mental health risks in older populations.

Furthermore, being overweight or obese was associated with a lower risk of psychological distress in our sample, consistent with previous research. This finding may lend support to the “jolly fat” hypothesis, suggesting that overweight or obese individuals exhibit fewer depressive symptoms and have a lower risk of depression [36, 40, 41]. This association might be particularly relevant in our study population, as Chinese traditional culture views having a good appetite as a blessing and obesity as a symbol of good social and economic status [42], which differs significantly from Western societies and younger populations where excess body weight is often stigmatized [43]. Although the hypothesis has been frequently tested, the results remain somewhat controversial, as some studies have reported no significant relationship between obesity and depression in longitudinal analyses [44]. Nevertheless, the long-term follow-up design of our study, which accounted for BMI changes, provides additional evidence supporting the protective role of being overweight in reducing psychological distress among older adults. A potential mechanism for this association may involve greater physical reserves contributing to improved well-being and lower depressive symptoms [40].

Our study also examined whether the association between BMI change and psychological distress differed by sex and age. Previous studies have reported inconsistent findings due to variations in study designs and populations [45, 46]. Some studies found sex-specific associations, such as increased depression risk in obese men and underweight or class II obese women [45], while others reported no significant effects [46]. However, these studies primarily focused on adults rather than on older adults and did not account for weight changes. Although one study on middle-aged to older adults linked weight loss > 3% to men, its limited sample size restricted its statistical power [15]. Our study demonstrated that weight loss was robustly and dose-dependently associated with psychological distress across sexes and age groups. The association with weight gain, observed in the repeated-measures analysis, was not robust in incident and lagged analyses, suggesting that it may partly reflect concurrent co-occurrence at a given examination rather than a prospective effect; weight loss, by contrast, appears to be the more consistent prospective marker of subsequent distress. Regarding age, the association was consistent across both the 65–79 and ≥ 80 years groups, underscoring the importance of stable body weight for mental health in older adults. These results provide robust evidence for the relationship between BMI changes and psychological distress across subgroups, addressing gaps in previous research.

The strengths of this study include its unique focus on examining the psychological impact of BMI changes in older adults, longitudinal design, large sample size, and adjustment for potential confounders. However, several limitations should be noted. First, the study participants were older adults from Taipei who voluntarily attended health examinations, limiting the generalizability of the findings to other populations or settings. Second, individuals with severe psychological distress may have been less likely to participate in health examinations, potentially leading to an underestimation of the association between BMI changes and psychological distress. Third, due to the observational nature of this study, causal relationships between BMI changes and psychological distress cannot be established. Future research should adopt more rigorous methodologies, such as experimental studies and randomized controlled trials, to better determine causality.

Several factors warrant caution in interpreting the associations. First, reverse causality is plausible: psychological distress can reduce appetite and food intake and thereby lead to weight loss, and subclinical or undiagnosed conditions, such as occult malignancy, frailty, or sarcopenia, may produce both weight loss and distress. To address this, we conducted lagged and incident (survival) analyses where weight change preceded the distress assessment; weight loss continued to predict distress in a dose-dependent manner, suggesting that the association is less likely to be fully explained by reverse causality, although the attenuated effect sizes suggest that part of the cross-sectional association reflects concurrent processes. Reverse causality nonetheless cannot be entirely excluded even in this prospective design. Second, although we adjusted for measured comorbidities, residual confounding by unmeasured factors (e.g., frailty, functional status and activities-of-daily-living dependence, cognitive decline, and undiagnosed chronic disease) may remain. Third, because height tends to decline with age, using per-visit height would attenuate the apparent magnitude of BMI loss, biasing our weight-loss estimates toward the null and rendering them conservative.

Significant BMI changes, whether short- or long-term, were associated with increased psychological distress in older adults, with weight loss posing a higher risk than weight gain. These findings suggest that weight changes may serve as early markers of underlying health issues. Clinically, regular monitoring of BMI changes, rather than static measurements, could help detect psychological distress early. Because weight can be routinely and inexpensively measured, a clinically meaningful change, particularly weight loss, could serve as a low-cost prompt for mental-health screening alongside nutritional and psychosocial assessment, supporting surveillance of weight trajectories rather than weight status alone. These findings support the need for targeted assessment when clinically meaningful weight change, particularly weight loss, is observed. Further research is needed to explore the causal pathways of this association.

## Supplementary Information

Supplementary Material 1.

## Data Availability

This study utilized data from the Taipei City Public Health Database, provided by the Department of Health, Taipei City Government, and managed by the Center for Public Health. Due to restrictions, the data cannot be made publicly available. However, researchers interested in accessing the data for replication purposes may contact the corresponding author for more details on the application process through the Taipei City Government.
