# Supplementary material for: Psychological impact of body mass index changes in older adults: a 12-year longitudinal study
Source: BMC Geriatr. 2026 Jul 29;26:1348. doi: 10.1186/s12877-026-08050-0 (PMC13700145; doi:10.1186/s12877-026-08050-0)
Supplement: Supplementary file 1 [file 12877_2026_8050_MOESM1_ESM.docx]

**Supplementary Material**

Supplementary Table S1 Sensitivity analysis: Cox proportional-hazards model for incident psychological distress (first BSRS-5 ≥ 6), with BMI change as a time-varying covariate, compared with the primary GEE model

Supplementary Table S2 Sensitivity analysis: lagged model relating BMI change at a given examination to psychological distress at the subsequent examination (restricted to participants without distress at the index examination), compared with the primary GEE model.

**Supplementary Table S1.** Sensitivity analysis: Cox proportional-hazards model for incident psychological distress (first BSRS-5 ≥ 6), with BMI change as a time-varying covariate, compared with the primary GEE model

| **BMI change category** | **Primary GEE, aOR (95% CI)** | **Cox HR (95% CI)** |
| --- | --- | --- |
| ***Definition 1 (change vs. baseline)*** | | |
| Major weight gain | 1.29 (1.18–1.41) | 0.85 (0.77–0.94) |
| Minor weight gain | 1.27 (1.20–1.35) | 0.91 (0.85–0.96) |
| Stable weight (reference) | 1.00 | 1.00 |
| **Minor weight loss** | 1.60 (1.52–1.68) | **1.16 (1.10–1.23)** |
| **Major weight loss** | 1.90 (1.77–2.04) | **1.25 (1.15–1.35)** |
| ***Definition 2 (change vs. previous visit)*** | | |
| Major weight gain | 1.51 (1.36–1.66) | 0.87 (0.77–0.98) |
| Minor weight gain | 1.38 (1.30–1.46) | 1.00 (0.93–1.07) |
| Stable weight (reference) | 1.00 | 1.00 |
| **Minor weight loss** | 1.52 (1.44–1.60) | **1.07 (1.00–1.14)** |
| **Major weight loss** | 1.96 (1.80–2.14) | **1.19 (1.08–1.31)** |

Notes. aOR, adjusted odds ratio; CI, confidence interval; HR, hazard ratio. The Cox model treated the first BSRS-5 ≥ 6 during follow-up as the event (10,840 events among 70,570 participants), with years since baseline (from examination dates) as the time scale and BMI change as a time-varying covariate. Both models were adjusted for baseline BMI, number of examinations, age, sex, marital status, education, living alone, low-income household, smoking, drinking, exercise, hypertension, diabetes, hyperlipidemia, and heart disease.

**Supplementary Table S2.** Sensitivity analysis: lagged model relating BMI change at a given examination to psychological distress at the subsequent examination (restricted to participants without distress at the index examination), compared with the primary GEE model

| **BMI change category** | **Primary GEE, aOR (95% CI)** | **Lagged model, aOR (95% CI)** |
| --- | --- | --- |
| ***Definition 1 (change vs. baseline)*** | | |
| Major weight gain | 1.29 (1.18–1.41) | 0.99 (0.88–1.11) |
| Minor weight gain | 1.27 (1.20–1.35) | 0.97 (0.91–1.04) |
| Stable weight (reference) | 1.00 | 1.00 |
| **Minor weight loss** | 1.60 (1.52–1.68) | **1.18 (1.11–1.26)** |
| **Major weight loss** | 1.90 (1.77–2.04) | **1.32 (1.19–1.45)** |
| ***Definition 2 (change vs. previous visit)*** | | |
| Major weight gain | 1.51 (1.36–1.66) | 1.23 (1.08–1.40) |
| Minor weight gain | 1.38 (1.30–1.46) | 1.08 (1.00–1.16) |
| Stable weight (reference) | 1.00 | 1.00 |
| **Minor weight loss** | 1.52 (1.44–1.60) | **1.12 (1.05–1.20)** |
| **Major weight loss** | 1.96 (1.80–2.14) | **1.29 (1.13–1.47)** |

Notes. aOR, adjusted odds ratio; CI, confidence interval. The lagged model used generalized estimating equations (binomial distribution, logit link, AR-1 working correlation) relating BMI change at each examination to psychological distress at the subsequent examination, among person-visits without distress at the index examination (13,377 incident transitions). Adjusted for the same covariates as in Supplementary Table S1.
